# Occurrence of 26 Mycotoxins in the Grain of Cereals Cultivated in Poland

**DOI:** 10.3390/toxins8060160

**Published:** 2016-05-25

**Authors:** Marcin Bryła, Agnieszka Waśkiewicz, Grażyna Podolska, Krystyna Szymczyk, Renata Jędrzejczak, Krzysztof Damaziak, Alicja Sułek

**Affiliations:** 1 Department of Food Analysis, Prof. Waclaw Dabrowski Institute of Agricultural and Food Biotechnology, Rakowiecka 36, 02-532 Warsaw, Poland; krystyna.szymczyk@ibprs.pl (K.S.); renata.jedrzejczak@ibprs.pl (R.J.); 2 Department of Chemistry, Poznan University of Life Sciences, Wojska Polskiego 75, 60-625 Poznań, Poland; agat@up.poznan.pl; 3 Institute of Soil Science and Plant Cultivation – State Research Institute, Department of Cereal Crop Production, Czartoryskich 8, 24-100 Puławy, Poland; aga@iung.pulawy.pl (G.P.); sulek@iung.pulawy.pl (A.S.); 4 Department of Poultry Science, University of Life Sciences, Ciszewskiego 8, 02-786 Warsaw, Poland; krzysiekkd12@wp.pl

**Keywords:** mycotoxins, grain, LC-TOF-HRMS, weather conditions, *Fusarium*

## Abstract

The levels of 26 mycotoxins were determined in 147 samples of the grain of cereals cultivated in five regions of Poland during the 2014 growing season. The HPLC-HRMS (time-of-flight) analytical technique was used. An analytical procedure to simultaneously determine 26 mycotoxins in grain was developed, tested and verified. Samples from eastern and southern Poland were more contaminated with mycotoxins than the samples from northern and western Poland. Toxins produced by *Fusarium* fungi were the main contaminants found. Some deoxynivalenol (DON) was found in 100% of the tested samples of wheat (Osiny, Borusowa, Werbkowice), triticale, winter barley and oats, while the maximum permissible DON level (as defined in the EU Commission Regulation No. 1881/2006) was exceeded in 10 samples. Zearalenone (ZEN), DON metabolites and enniatins were also commonly found. The presence of mycotoxins in grain reflected the prevailing weather conditions during the plant flowering/earing stages, which were favorable for the development of blight. Among all investigated wheat genotypes, cv. Fidelius was the least contaminated, while Bamberka, Forkida and Kampana were the most contaminated. However, the single-factor ANOVA analysis of variance did not reveal (at a statistical significance level α = 0.05) any differences between levels of mycotoxins in individual genotypes. Triticale was the most contaminated grain among all of the tested varieties. ZEN, DON and the sum of 3-acetyldexynivalenol and 15-acetyldeoxynivalenol (3- and 15-ADON) were found in 100% of the tested triticale samples at concentrations within the 4–86, 196–1326 and 36–374 µg·kg^−1^ range, respectively. Of particular concern was the fact that some “emerging mycotoxins” (enniatins) (in addition to commonly-known and legally-regulated mycotoxins) were also found in the tested triticale samples (enniatin B (Enn-B), enniatin B1 (Enn-B1), enniatin A-1 (Enn-A1), 100% of samples, and enniatin A (Enn-A), 70% of samples). Depending on the toxin, they were found at levels between 8 and 3328 µg·kg^−1^.

## 1. Introduction

Generally, mycotoxins are toxic metabolites of various fungi found in various plant-origin food around the world. Several toxins may be found in a single foodstuff. Mycotoxins may negatively impact consumer health and are therefore regarded as a serious threat to food safety [1]. The economic consequences are rather serious: it is estimated that approximately 25% of global production of grain (approximately 20% of the total production of plant-origin food) is contaminated with mycotoxins. Of course, the level of contamination depends significantly on local conditions [2]. In addition, mycotoxins in food/feed generally constitute a serious threat for food safety.

Food safety concerns are increasing due to the potential consequences of climate changes that might influence crop yield and the degree to which the crops are contaminated with mycotoxins. Climate changes might have an enormous impact on landscapes around the world. A significant increase in the average temperature and a decrease in rainfall during the summer season are predicted for some regions. Weather conditions may be favorable for the development of fungi that have not yet been observed within a given area [3,4].

Agriculture contributes significantly to the value of the national economy in Poland. Plant production accounts for approximately 43% of total agricultural production, while cereals make up approximately 37% of the plant production. In 2013, Polish farmers produced approximately 9.5 million tons of wheat, 3.4 million tons of rye, 2.9 million tons of barley, 1.2 million tons of oats and 4.3 million tons of triticale. Poland is ranked second (after France) in the EU with respect to arable land and third (after France and Germany) with respect to crop volume. Even though crop yield and total production of cereals in Poland fluctuate on an annual basis, reflecting changes in weather conditions, we may observe a clear upward long-term trend in crop yield, indicating a clear increase [5].

Mycotoxins, for which maximum levels in grain for food have been regulated, include aflatoxin B_1_, the sum of B_1_/B_2_/G_1_/G_2_ aflatoxins, zearalenone, deoxynivalenol, ochratoxin A (OTA) and the sum of B_1_/B_2_ fumonisins [6,7,8]. Moreover, indicative levels have been recommended for HT-2 and T2 toxin concentrations in cereals [9]. In grain for feed, an acceptable level has been specified only for aflatoxin B_1_ [10], and limits have been recommended for OTA, DON, ZEN and B_1_/B_2_ fumonisins [11]. However, food/feed may be contaminated with a much wider range of mycotoxins. The European Food Safety Authority has been evaluating various mycotoxins in food and feed since 2008. The list of substances for which toxicological risk should initially be evaluated includes type B trichothecenes (DON and its derivatives, nivalenol (NIV)), type A trichothecenes (HT-2 and T-2 toxins, diacetoxyscirpenol (DAS)), fumonisins, enniatins (Enns), beauvericin (BEA), toxins of *Alternaria* fungal, ergot alkaloids, patulin, citrinin, sterigmatocystin, moniliformin (MON) and phomopsin [12].

Even if analyses of mycotoxins in food are typically difficult due to low concentrations of the analytes and the high complexity of the matrices [13], a large number of methods are able to determine both individual mycotoxins and/or groups of substances of a similar structure in food/feed [1,2,9]. These immunologic or GC/LC-based methods use a variety of detectors. Validated methods capable of simultaneously detecting many mycotoxins/groups of mycotoxins are important elements of every program that monitors mycotoxins in grain for food/feed, in other agricultural produce and/or in processed food [1]. Currently, mycotoxins in food/feed are increasingly determined by means of a high performance liquid chromatograph coupled with a mass spectrometer. Advances in the technique have made it possible to fine-tune it, so that some analytical methods are now capable of simultaneously determining many mycotoxins in various food matrices (multi-analyte methods). Efficient sample preparation is the main challenge faced during the optimization of any of the above-mentioned analytical methods. The technique used to extract numerous analytes from various matrices should yield high recovery rates to enable low limits of determination and should have good repeatability (precision). In a majority of available analytical methods, certain water/acetonitrile mixtures are used to extract analytes. Other techniques also employed in many methods include solid phase extraction, liquid-liquid extraction (fat removal using hexane) and immunoaffinity columns. Sample purification performed with the objective of limiting the matrix effects may simultaneously restrict the range of analytes that the method is capable of determining and may decrease the recovery rates [14].

The aims of this work were: (i) to develop/test/verify an HPLC-HRMS analytical procedure capable of simultaneously determining many various mycotoxins in grain; (ii) to apply the developed method to determine the levels of mycotoxins in various grains (including various genotypes of winter wheat) cultivated in different regions of Poland; and (iii) to correlate the measured mycotoxin levels in various wheat genotypes and with weather conditions during the grain growing season. This is one of the first scientific reports on the natural occurrence of certain mycotoxins (e.g., masked mycotoxins and enniatins) in cereals grown in Poland.

## 2. Results and Discussion

### 2.1. Weather Conditions/Wheat Genotype

**Figure 1:**
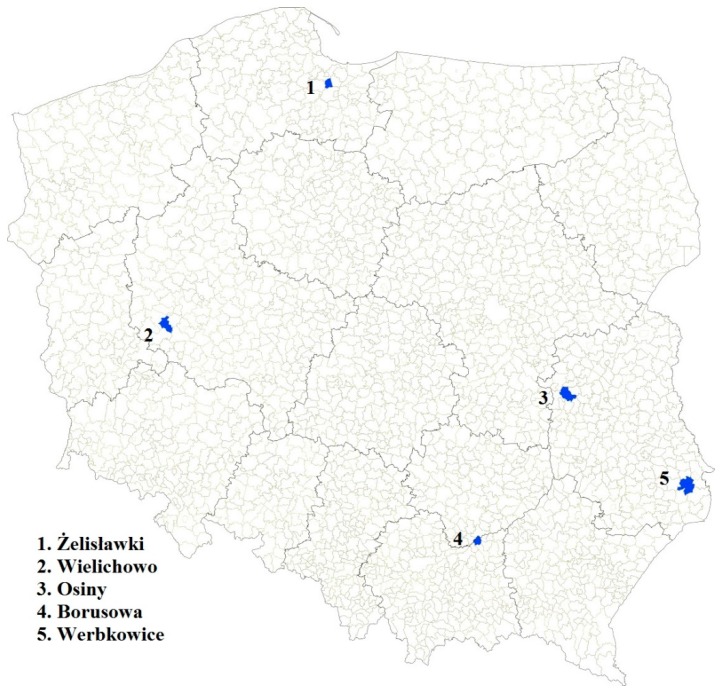
Five regions of Poland in which samples for this study were cultivated. 1. Żelisławki (54°09′32″ N 18°39′02″ E), 18 wheat samples (two samples of each cultivar); 2. Wielichowo (52°07′08″ N 16°20′58″ E), 36 wheat samples (four samples of each cultivar); 3. Osiny (51°27′53″ N 22°03′52″ E), 18 wheat samples (two samples of each cultivar); 4. Borusowa (50°16′39″ N 20°48′02″ E), 18 wheat samples (two samples of each cultivar), 5. Werbkowice (50°45′06″ N 23°46′57″ E), nine wheat samples (one sample of each cultivar).

A total of 147 samples of cereals cultivated in five regions of Poland were analyzed (Figure 1). The samples included 99 samples of nine winter wheat cultivars (Astoria, Bamberka, Dacanto, Fidelius, Forkida, Kampana, Kepler, Meister, Oxal).

**Figure 2:**
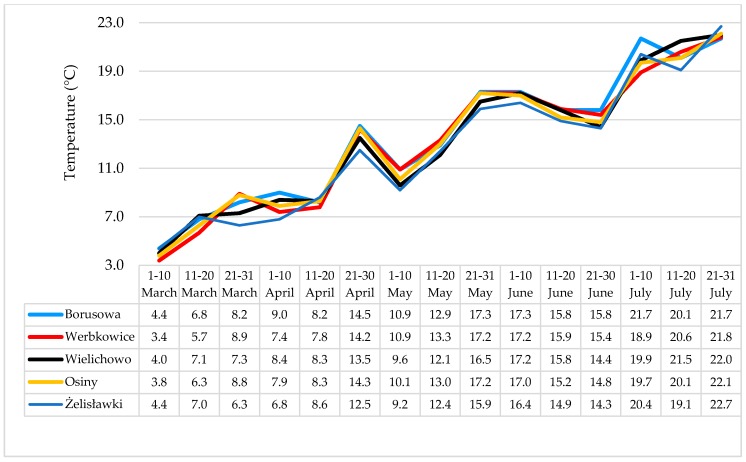
Ten-day average temperatures (°C) during the wheat vegetation period in 2014.

**Figure 3:**
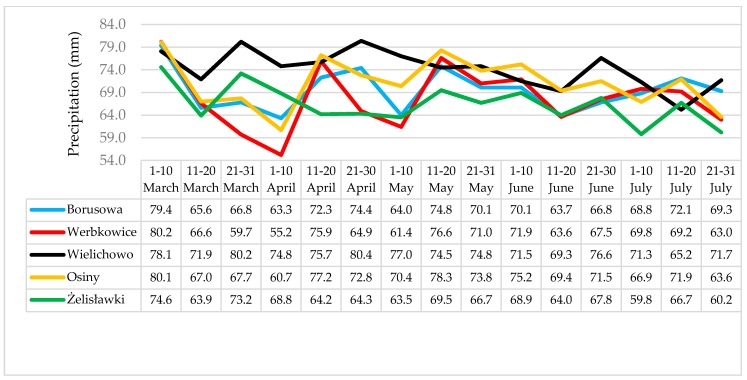
Ten-day average rainfall levels (mm) during the wheat vegetation period in 2014.

The communes of Osiny, Borusowa and Werbkowice (situated relatively close to one another) are located in the southeastern part of Poland, with Żelisławki in the north and Wielichowo in the west. The air temperatures and rainfall were monitored at each location from March to July 2014, *i.e.*, until the end of the grain ripening period. In Figure 2 and Figure 3, the trends in the prevalence of temperature and rainfall in different localities is shown. In general, the temperatures recorded at Żelisławki were lower than at the other locations (average 7.6 °C in March and April compared to 8.1 °C for the other locations; 16.2 °C in May, June and July (flowering/earing season) compared to 17.1 °C for the other locations). During the wheat earing stage, the greatest rainfall and temperature values were recorded in Osiny, Borusowa and Werbkowice; the respective values during the second period of May were as follows: rainfall, 78.3, 74.8 and 76.6 mm; temperature, 13.0, 12.9 and 13.3 °C; and the respective values for May during the third period were 17.2, 17.3 and 17.2 °C (compared to 16.5 °C in Wielichowo and only 15.9 °C in Żelisławki). Some spikelets of crops cultivated in Osiny, Borusowa and Werbkowice showed whitening during the second period, which resulted in the development of ear blight (FHB).

During the wheat flowering period, rainfall was lower in Żelisławki than in Osiny, Werbkowice and Borusowa by 11.2 mm, 7.9 and 6.1 mm, respectively. The rainfall was also lower in Wielichowo by 5.9, 2.6 and 0.8 mm, respectively. Average temperatures were lower in Żelisławki than in the three other locations by 2.1, 2.3 and 2.4 °C, respectively. The temperatures were also lower in Wielichowo by 1.2, 1.4 and 1.5 °C, respectively. Lower temperatures and lower air humidity must have limited the growth of *Fusarium* fungi, hence mycotoxin biosynthesis. Czaban *et al.* [15] reported a strong positive correlation between maximum temperature/percentage of days with maximum temperatures not lower than 15 °C and the percentage of wheat kernels infected by *F. graminearum* (*r* = 0.976/0.996), *F. culmorum* (*r* = 0.998/0.981) and *F. tricinctum* (*r* = 0.879/0.933), respectively. Franz *et al.* [16] found that DON concentration is positively correlated with the number of hours with relative humidity (RH) > 90%.

**Figure 4:**
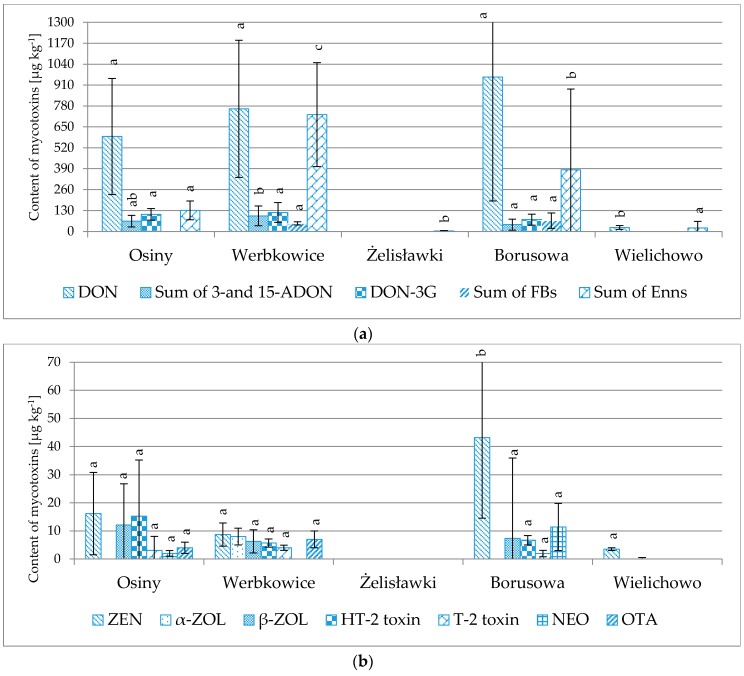
Comparison of average concentrations of individual mycotoxins (µg·kg^−1^) in winter wheat cultivated in Osiny, Borusowa and Werbkowice. Different letters mark different homologous groups. (**a**) for DON, Sum of 3-and 15-ADON, DON-3G, Sum of FBs, Sum of Enns; (**b**) for ZEN, α-ZOL, β-ZOL, HT-2 toxin, T-2 toxin, NEO, OTA.

As observed in Table 1 and Figure 4, the winter wheat cultivated in Osiny, Borusowa and Werbkowice was more highly contaminated than the wheat cultivated in Żelisławki and Wielichowo. The following *Fusarium* toxins were found most frequently in the former samples: DON, ZEN, Enn-B, Enn-B1 (found in all analyzed samples), Enn-A1 (found in 89%–100% of samples), T-2 (78%–100%), Enn-A (56%–100%), β-ZOL (44%–100%), 3-/15-ADON (67%–83%), DON-3G (44%–78%), HT-2 (17%–67%), and FB_1_ (6%–22%). Neosolaniol (NEO) was only found in a single sample from Osiny and in 15 samples from Borusowa. The highest concentrations (in µg·kg^−1^) found in the samples cultivated in Osiny, Borusowa and Werbkowice included: DON 209–2,975 (average 960); Enn-B 20–1,981 (average 275); DON-3G 40–356 (average 118); DON-3G 43–166 (average 106); Enn-B1 8–368 (average 89); 3-/15-ADON 32–98 (average 59); and ZEN 7–100 (average 43). Wheat cultivated in Żelisławki and Wielichowo was contaminated with mycotoxins to a much lesser extent. Among the Wielichowo samples, ZEN was found in two cases (3–4 µg·kg^−1^), DON in only a single sample (25 µg·kg^−1^), Enn-B in 30 samples (1–156 µg·kg^−1^) and Enn-B1 in 23 samples (1–22 µg·kg^−1^). Among the Żelisławki samples, Enn-B was found in 18 cases and Enn-B1 in 15 samples; however, the measured mycotoxin levels were low (1–7 µg·kg^−1^). The maximum acceptable levels of mycotoxins in grain (as defined in the EU Commission Regulation No. 1881/2006 [6]) were exceeded in eight cases: the 1250 µg·kg^−1^ DON level (for unprocessed grain) was exceeded in seven samples (one from Osiny, five from Borusowa, one from Werbkowice), while the 100 µg·kg^−1^ ZEN (level for unprocessed grain) level was exceeded in a single sample from Osiny.

It is possible that local weather conditions prevailing during the wheat blossoming period are responsible for the fact that the wheat cultivated in Żelisławki and Wielichowo was clearly less contaminated with mycotoxins than that cultivated in Osiny, Borusowa and Werbkowice. Cereal plants cultivated in a temperate climate are frequently infected with *Fusarium* fungi [17]. The varieties often encountered in Europe include *F. poae*, *F. tricinctum*, *F. avenaceum*, *F. culmorum* and *F. graminearum* [18]. *F. avenaceum*, *F. culmorum* and *F. graminearum* are the main pathogens of wheat in Poland [19]. Lukanowski and Sadowski [20] have also isolated *F. langsethiae* from winter wheat. *Fusarium* spores in wheat grain are rather ubiquitous. Factors influencing the development of fusariosis include other crops cultivated in close vicinity and/or residues of maize left on the soil surface after harvesting [21]. Pathogens overwinter inside the lignified remains of maize and become a source of inoculum at a later stage of plant development [22]. Rainfall is a commonly-used factor in predicting grain vulnerability to diseases. Research conducted in numerous countries has shown that moderate temperatures (15–30 °C) combined with prolonged periods of high humidity during the blossoming and/or earing phases promote the accumulation of DON and are the best indicators of fusariosis [23,24,25].

**Figure 5:**
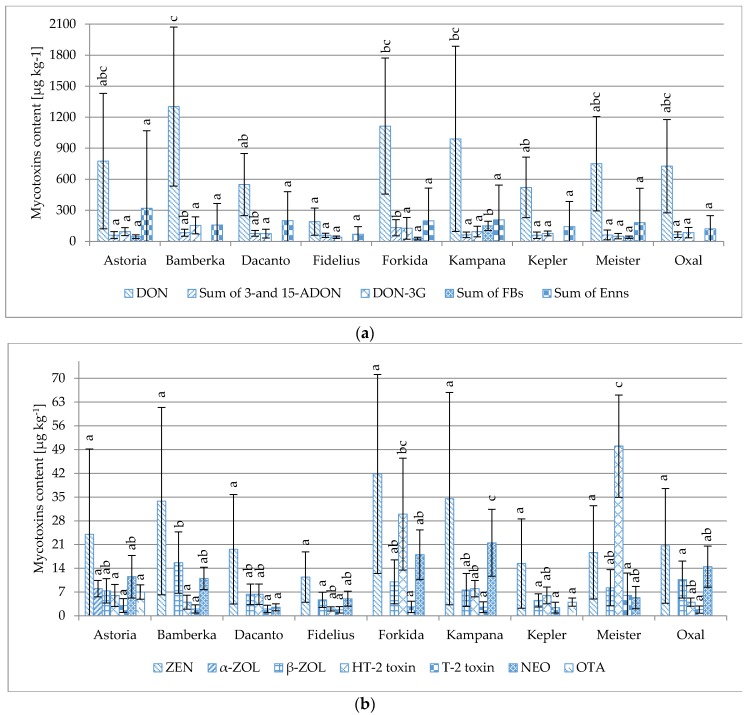
Comparison of the average concentrations of individual mycotoxins (µg·kg^−1^) in nine studied genotypes of winter wheat cultivated in Osiny, Borusowa and Werbkowice. Different letters mark different homologous groups. (**a**) for DON, Sum of 3-and 15-ADON, DON-3G, Sum of FBs, Sum of Enns; (**b**) for ZEN, α-ZOL, β-ZOL, HT-2 toxin, T-2 toxin, NEO, OTA.

The same genotypes of winter wheat were cultivated in all five regions. Significant contamination with mycotoxins (mostly produced by *Fusarium* fungi) was observed in wheat cultivated in Osiny, Borusowa and Werbkowice. The single-factor ANOVA analysis of variance did not reveal significant differences between levels of majority of mycotoxins in the tested genotypes of wheat. DON is an exception: significant differences were found between the Fidelius and Kampana/Forkida/Bamberka varieties, as well as between the Kepler/Dacanto and Bamberka ones; see Figure 5. Depending on the genotype, the lowest contamination was found in cv. Fidelius (in µg·kg^−1^) (DON, 190; ZEN, 11; sum of 3-/15-ADON, 59; DON-3G, 40, sum of Enns, 69; ß-ZOL, 5; HT-2 toxin, 2; T-2 toxin, 2; NEO, 5) to the most contaminated cv. Bamberka (DON, 1302; ZEN, 34; sum of 3-/15-ADON, 132; DON-3G, 155; sum of Enns, 159; ß-ZOL, 16; HT-2 toxin, 4; T-2 toxin, 2; NEO, 11).

The selection of genotypes resistant to fusariosis (FHB) is one of the methods used in wheat cultivation to restrict the incidence of mycotoxins in wheat grain. However, none of the tested genotypes have proved fully immune [26,27]. FHB resistance is controlled by major and minor genes located on all wheat chromosomes, except 7D [28]. Sumai 3, a Chinese spring wheat cultivar (with the *QTL* and *Fhb1* genes), is commonly regarded as the most resistant genotype and is widely applied in breeding programs around the world [29,30]. Studies on increasing the resistance of wheat to FHB are underway. The recently-generated winter wheat cv. Truman may be an example of a positive effect of such studies, as nearly perfect resistance to FHB was obtained [30]. However, all currently registered winter wheat varieties are more or less susceptible to FHB. When artificially infected with *F. culmorum*, most cultivars prove susceptible or very susceptible to ear fusariosis compared to commonly-known resistant cultivars, e.g., Arina [26]. Data collected at the Polish Research Centre for Cultivar Testing (Centralny Ośrodek Badania Odmian Roślin Uprawnych—COBORU) in Słupia Wielka for all of the tested varieties showed very similar resistance to FHB (ranging from 7.2 (Fidelius, Forkida) to 7.9 (Meister) in the COBORU 1–9 scale), where 1 is very susceptible and 9 is resistant. Our results are consistent with the COBORU findings only in the case of the Fidelius cultivar. Such a lack of correlation in the case of most cultivars suggests that there are additional factors that contribute to the accumulation of mycotoxin in the kernels. For example, tiller height plays a role in the sensitivity of the cereals to FHB. Mikos-Szymańska and Podolska [31] reported significant negative correlation between triticale height and mycotoxin contamination. Similarly, Choo *et al.* [32] reported a significant negative correlation between barley height and DON content. Ransom and McMullen [33] suggested that the shorter cultivars, exhibiting stronger FHB severity, tended to be closer to the inoculum source, which probably explains why the taller varieties were less affected. Another factor that most likely contributes to differences in mycotoxin accumulations is the structure and composition of the kernels. For example, Walter *et al.* [34] reported that the plant cuticle, middle lamellae and cell walls are important physical barriers that fungal intruders have to overcome. Additional morphological features, such as grain coloration with phenols, waxiness and the concentration of ferulic acid, are also believed to modulate mycotoxin level in cereals [15].

### 2.2. Other Cereal Cultivars

**Figure 6:**
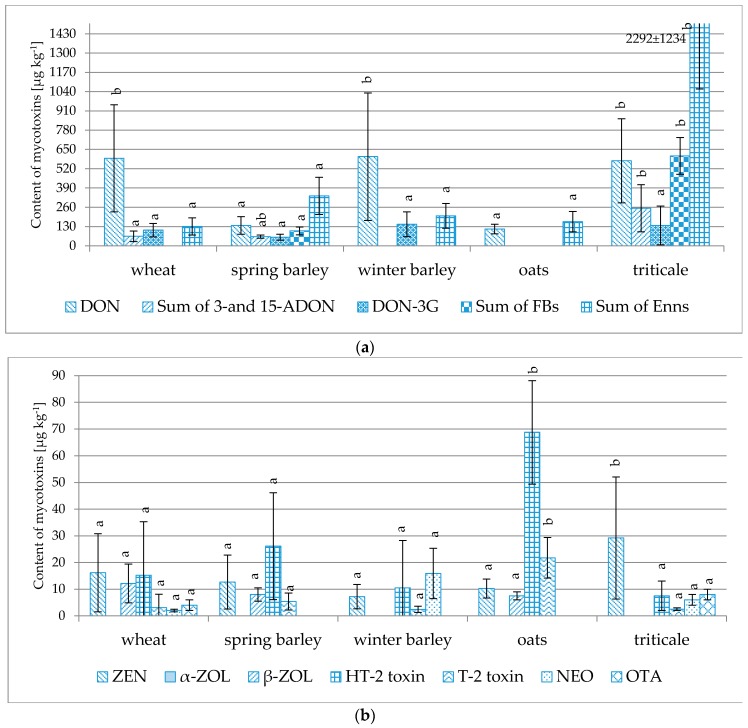
Comparison of the average concentrations of individual mycotoxins (µg·kg^−1^) in various cereals cultivated in Osiny. Different letters mark different homologous groups. (**a**) for DON, Sum of 3-and 15-ADON, DON-3G, Sum of FBs, Sum of Enns; (**b**) for ZEN, α-ZOL, β-ZOL, HT-2 toxin, T-2 toxin, NEO, OTA.

Grain sampled at Osiny also included eight samples of spring barley, 16 samples of winter barley, four samples of oats and 20 samples of triticale. As observed in Table 2, among these samples, triticale was the grain that was most frequently contaminated with *Fusarium* toxins, mostly DON, ZEN, 3-/15-ADON, Enn-B, Enn-B1, Enn-A (found in all 20 analyzed samples), Enn-A1 (89%–100% of samples) and Enn-A1 (70%). The highest concentrations (in µg·kg^−1^) included: Enn-B 473–3328 (average 1360); DON 196–1326 (average 573); Enn-B1 149–1347 (average 552); Enn-A1 67–882 (average 357); 3-/15-ADON 36–374 (average 162); Enn-A 8–135 (average 32); and ZEN 4–86 (average 29). Maximum acceptable levels were exceeded in two samples: an excessive concentration of DON was found in a single sample, and another single sample contained OTA at a concentration of 8 µg·kg^−1^, *i.e.*, above the 5 µg·kg^−1^ level specified in the EU Commission Regulation No. 1881/2006 [6]. The mycotoxins found in winter barley included DON, Enn-A1, Enn-B, Enn-B1 (found in all 16 analyzed samples), HT-2 (75% of samples), ZEN (62.5%), T-2 (44%), NEO (44%) and DON-3G (32.5%). The highest concentrations (in µg·kg^−1^) were as follows: DON 54–1,602 (average 602); Enn-B 49–253 (average 150); DON-3G 43–277 (average 146); and Enn-B1 8–81 (average 46). Maximum acceptable DON levels were exceeded in two samples. Spring barley and oats were contaminated to a much lesser extent. The significance of the differences between the levels of the individual compounds in the tested samples is shown in Figure 6.

Mycotoxins are commonly found in cereals around the world. In Poland, mycotoxins produced by *Fusarium* fungi are the main threat to food/feed safety. Studies on the AF, OTA, DON, NIV, HT-2, T-2, ZEN and FB mycotoxins in fodder based on grain (including maize) cultivated between 2006 and 2009 in various regions of Poland identified DON, ZEN, OTA, T-2 and HT-2 as the most frequently-occurring toxins [35]. The percentages of positive samples and the average concentrations were as follows: DON 71%–90% of samples, 38–288 µg·kg^−1^; ZEN 45%–74%, 1.20–6.22 µg·kg^−1^; OTA 26%–88%, 1.99–33 µg·kg^−1^; T-2 23%–52%, 3.9–5.5 µg·kg^−1^; and HT-2 30%–67%, 9.6–11.8 µg·kg^−1^ (depending on the grain vegetation season).

*Fusarium* toxins (DON, ZEN, HT-2, T-2) were also identified in *Triticum aestivum* wheat cultivated in Lithuania during the 2010–2012 seasons [36]. Some grain was produced under environmentally-sound conditions. Depending on the season, the DON concentrations were low (<100 µg·kg^−1^ in 2010), moderate (95% positive samples, average concentration 183.8–990.9 µg·kg^−1^ in 2011) or high (100% positive samples, 115.5–8845.1 µg·kg^−1^ in 2012). These findings correlate well with the fact that intense rainfall combined with high average air temperatures prevailed in the third decade of July 2012. The ZEN concentrations were 11.0–12.7 µg·kg^−1^ (2010), 11.6 µg·kg^−1^ (only a single ZEN-positive sample in 2011) and 17.2–302.5 µg·kg^−1^ (2012), and the HT-2 + T-2 toxin concentrations were 10.2–24.6 µg·kg^−1^ (2010), 10.0–115 µg·kg^−1^ (2011) and 10–23.7 µg·kg^−1^ (2012).

Edwards *et al.* [37] reported that 92%/84% of samples of oats cultivated between 2002 and 2005 in the U.K. were contaminated with HT-2/T-2, respectively (the average concentration of the sum of both toxins was 570 µg·kg^−1^). Similar results were reported by Pettersson *et al.* [38] for oats cultivated in Sweden and Scudamore *et al.* [39] (oats cultivated in the U.K.). These toxins are produced by *F. langsethiae*.

The so-called “masked” mycotoxins (including DON/ZEN metabolites) are becoming increasingly important. However, there are very few reports on their occurrence in Polish cereal grains. Globally, DON-3G is the most frequently identified among masked mycotoxins in wheat grain [40]. Consequently, data on this mycotoxin are the most readily available among all data on masked mycotoxins [41]. The highest DON-3G levels in naturally-contaminated crops were found in wheat samples: above 1000 µg·kg^−1^, in 20%–70% relative to DON [42,43,44]. We found DON-3G in all of our positive samples, at a proportion of 2%–48% relative to DON; the triticale samples were among the most contaminated (40–434 µg·kg^−1^).

Reports on the occurrence of enniatins in Polish cereal grains are equally sparse. Globally, the levels seem to depend on the particular climatic zone. Oueslati *et al.* [45] analyzed enniatins in 51 samples of wheat, barley, maize and sorghum cultivated in Tunisia. Enniatins were found in all analyzed wheat samples (at average concentrations of 48.9/90.2/75.3/30.6 mg kg^−1^ for Enn-A/A1/B/B1, respectively). Enniatins were also found in oats/barley/wheat cultivated in Norway between 2000 and 2002 [46]. The percentages of positive samples found by these authors were as follows: Enn-A 25%; Enn-A1 67%; Enn-B 100%; Enn-B1 94%. The average concentrations were Enn-A 5.8 µg·kg^−1^, Enn-A1 22 µg·kg^−1^, Enn-B 790 µg·kg^−1^ and Enn-B1 180 µg·kg^−1^ (for wheat), Enn-A <3 µg·kg^−1^, Enn-A1 6 µg·kg^−1^, Enn-B 47 µg·kg^−1^ and Enn-B1 20 µg·kg^−1^ (for oats) and Enn-A <4.5 µg·kg^−1^, Enn-A1 35 µg·kg^−1^, Enn-B 490 µg·kg^−1^ and Enn-B1 170 µg·kg^−1^ (for barley). Alkadri *et al.* [47] showed that the development of various fungi capable of producing various mycotoxins in grain depends primarily on the climate conditions prevailing during grain cultivation. Enniatins are also found in cereals. Malachova *et al.* [48] reported the following percentages of positive cereal samples and average concentrations: Enn-A 97%, 20–2532 µg·kg^−1^; Enn-A1 44%, 8–851 µg·kg^−1^; Enn-B 91%, 13–941 µg·kg^−1^; and Enn-B1 80%, 8–785 µg·kg^−1^.

The risk of fumonisin contamination of wheat, barley, oat and/or triticale is rather low due to the known inclination of *F. verticillioides* and *F. proliferatum* to infect maize [49]. Our analyses have revealed FB_1_ at levels between 15 and 150 μg kg^−1^ in six wheat samples, 101 μg kg^−1^ in one barley sample and 342 μg kg^−1^ in one triticale sample. The same triticale sample contained 151 and 113 μg kg^−1^ of FB_2_ and FB_3_, respectively. Even if the levels are relatively low, fumonisins in those grains are of concern, since so far, they contaminated mainly maize and rice [50], but were practically not found in other Polish cereal grains, in particular in wheat [36]. As much as 97% of 55 samples of Durum wheat cultivated in Argentina were however contaminated with FB_1_ + FB_2_ at levels between 10.5 and 1245.7 μg kg^−1^ [51]. Significantly lower contamination of samples cultivated in 2008 than in 2007 was attributed to different rainfall volume during both vegetation seasons. FB_1_ + FB_2_ fumonisins demonstrated in cultivated Spain cereals (72% positive samples of barley, concentration range 200–11,600 μg kg^−1^, 47% positive samples of wheat, concentration range 200–8,800 μg kg^−1^) were attributed to *F. proliferatum* [52]. Fumonisins were detected also in cereal grains cultivated in Iran: FB_1_/FB_2_/FB_/3_ was found in 68%/43%/32% of wheat samples at levels of 15–155/12–86/below 100 μg kg^−1^, respectively. *Fusarium* strains responsible for the biosynthesis of fumonisins were isolated from the grain [53].

OTA and OTB were found in only one of our triticale samples (the same sample). However, the OTA/OTB LOQ in our method was fairly high (4 µg·kg^−1^). Regardless of the climatic zone, the presence of OTA in grain reflects an inappropriate method of grain storage. In tropical zones and/or warm climates, OTA may be synthesized by *Aspergillus* fungi, compared to *Penicillium* *verrucosum* in moderate/cold zones. Aflatoxins are not the most significant contaminants of grain in Poland, although inappropriate grain storage may result in such contamination [54]. We found no AFs in our samples, but the LOQs of AFs in our method were also fairly high (5 μg kg^−1^). However, the analyzed grain was not sampled from heaps or storage bins, and the fact that no AFs were found may merely reflect that no AFs-producing fungi developed in the field.

**Figure 7:**
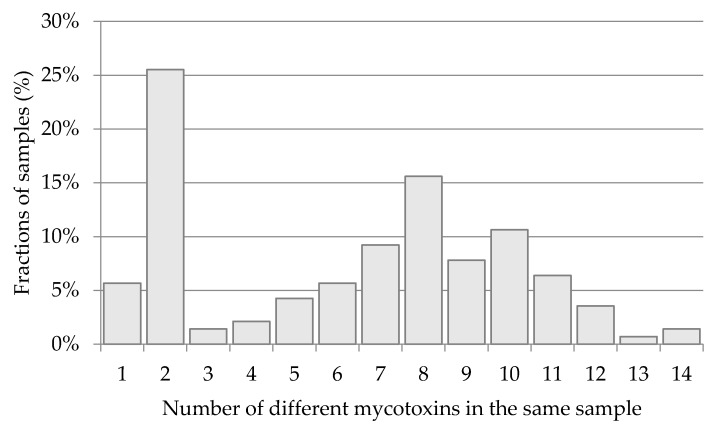
Distribution of the number of mycotoxins found in all positive samples.

The investigated samples were often contaminated with more than one mycotoxin. As observed in Figure 7, as much as 25% of all positive samples (including winter wheat samples) were contaminated with two (out of 26) investigated toxins; only 6% of samples were contaminated with individual toxins. At least three toxins were found in each of the remaining 69% of samples, among which the mycotoxin count peaked at eight. It was noted that DON is frequently accompanied by enniatins and/or other so-called “emerging mycotoxins” [55]. Little is known about the possible synergy of various mycotoxins acting in combination, and it is difficult to assess their real threat to health. However, such combinations may potentially be a serious toxicological problem [56,57].

## 3. Conclusions

A high performance liquid chromatography coupled with a (time-of-flight) mass spectrometer was employed to analyze levels of mycotoxins in wheat/barley/oat/triticale grain. An analytical procedure to simultaneously determine 26 mycotoxins in grains was developed, tested and verified.

The levels of 26 mycotoxins were determined in 99 samples of nine various genotypes of winter wheat (Astoria, Bamberka, Dacanto, Fidelius, Forkida, Kampana, Kepler, Meister, Oxal) cultivated in five regions of Poland during the 2014 growing season and in 48 samples of three other cereals (barley, oat, triticale) cultivated in the southeastern part of Poland (also during the 2014 growing season). Mycotoxins produced by *Fusarium* fungi were the major contaminants identified in the studied grain samples. The wheat cultivated in the southeastern part of Poland was more highly contaminated with mycotoxins than that cultivated in the northern/western regions of Poland, most likely as a result of the unfavorable weather conditions (considerable rainfall and high temperatures) prevailing during the plant flowering/earing stages in southeastern Poland. Among the grain of cereals other than winter wheat, triticale was the grain most often contaminated with *Fusarium* toxins. Spring barley and oats were contaminated to a much lesser extent. Among all of the investigated wheat genotypes, cv. Fidelius was the least contaminated, while Bamberka, Forkida and Kampana were the most contaminated.

The maximum acceptable DON level (1250 µg·kg^−1^) was exceeded in 10 samples (out of 147 total), while the maximum acceptable OTA level (5 µg·kg^−1^) was exceeded in a single sample. The fact that some enniatins (“emerging” mycotoxins that have not yet been legally regulated) were also found in all tested triticale samples (at 8–3328 µg·kg^−1^, depending on the mycotoxin) is cause for concern. The investigated samples were often contaminated with more than one single mycotoxin. As much as 25% of all positive samples (including winter wheat samples) were contaminated with two (out of 26) of the investigated toxins; only 6% of the samples were contaminated with a single toxin. At least three toxins were found in each of the remaining 69% of samples, among which the mycotoxin count peaked at eight. Little is known about the possible synergy of various mycotoxins acting in combination, and it is difficult to assess their real threat to health. However, such combinations may potentially be a serious toxicological problem.

## 4. Experimental Section

### 4.1. Samples

A total of 147 samples of various cereals cultivated in five regions of Poland were analyzed. The number of samples sampled in individual locations is shown in Table 3. The winter crops were sown exactly the same as winter wheat, whereas the spring crops were sown at the beginning of April 2014. The grain was sown, cultivated and harvested exactly the same as winter wheat.

Experimental plots (each covering an area of 36.0 m^2^) were established using a randomized blocks method with 3 replications. Wheat was sown in October 2013 (an optimal date for the region), in soil classified as a good wheat complex. The previous crop was winter rape. The seeding density was 450 sprouted seeds per sqm. Before sowing, the soil was sampled to determine the contents of available forms of macro-elements. According to the test results, NPK fertilizer (8-20-30) was used in the following amounts: 20 kg·N, 70 kg·P and 100 kg·K. Fertilizer (120 kg·N·ha^−1^) was applied during the growing season (50 kg at the start of the vegetation period, 40 kg at stalk shooting and 30 kg during earing). The land plots were protected against pathogens. A Pilmet 100 sprayer was used to perform the spraying operations. In autumn, 1 L·ha^−1^ of an Alister Granade preparation was used to control weeds; 2 L·ha^−1^ of Adexar Plus were used to control fungi; and 0.1 L·ha^−1^ of Furry 100 EW was used to control pests. Crops were harvested during the full ripeness stage using a small harvester.

Temperatures and rainfall during the plant vegetation period were monitored by weather stations located close to the experimental plots. Individual grain cultivars/genotypes were harvested separately using a small harvester. Grain harvested in blocks was formed into separate heaps. Samples for analyses were acquired directly after the harvest from heaps composed of grain originating from three blocks. To avoid secondary development of mold during storage, the moisture content of the harvested grain was maintained below 14%. Grain samples (2 kg each) were collected from various points in the heap using a pipe sampler (outer dimensions 40 × 2 mm, inner dimensions 35 × 3 mm, length 1,800 mm, 12 openings of 100 × 23 mm each; ZBPP, Bydgoszcz, Poland). The samples were ground using an MLU-202 laboratory mill by Bühler GmbH (Uzwil, Switzerland), and stored in a dry and cold location. Each analysis was performed in 3 independent replicates.

### 4.2. Reagents and Analytical Standards

The reagents used in this study included: HPLC-grade and LC-MS-grade methanol; HPLC-grade and LC-MS-grade acetonitrile (Rathburn Chemicals Ltd., Walkerburn, UK); LC-MS-grade water (Merck, Darmstadt, Germany); HPLC-grade and LC-MS-grade formic acid; sodium citrate dibasic sesquihydrate; sodium citrate dihydrate; PSA bonded on spherical silica; C-18 silica gel; LC-MS-grade ammonium formate (Sigma Aldrich, St. Louis, MO, USA); analytical grade magnesium sulfate (POCH, Gliwice, Poland); and neutral alumina (Merck, Darmstadt, Germany). HPLC-grade water used to extract the analytes was produced in-house using a Hydrolab demineralizer (Hydrolab, Straszyn, Poland).

Analytical standards purchased in the form of ready-to-use solutions from Romer Labs (Tulln, Austria) included: Mix 9 aflatoxins (AFB_1_, AFB_2_, AFG_1_, AFG_2_), 1 µg·mL^−1^ each; α-zearalenol (α-ZOL), β-zearalenol (β-ZOL), ochratoxin A (OTA), ochratoxin B (OTB). 10 µg·mL^−1^ each; Mix 3 fumonisins (FB_1_, FB_2_), deoxynivalenol-3-glucoside (DON-3G), hydrolysed fumonisin B_1_ (HFB_1_), fumonisin B_3_ (FB_3_), 50 µg·mL^−1^ each; HT-2, T-2, deoxynivalenol (DON), fusarenon X (FUS-X), neosolaniol (NEO), diacetoxyscirpenol (DAS), zearalenone (ZEN), 100 µg·mL^−1^ each; as well as 13C-labelled internal standards of ^13^C ZEN, DAS, HT-2, FB_1_: 25 µg·mL^−1^ each; and FB_2_, FB_3_: 10 µg·mL^−1^ each. Depending on solubility, the standards were dissolved in acetonitrile or in an acetonitrile/water mixture. Analytical standards purchased from Sigma Aldrich (St. Louis, MO, USA) and dissolved in-house in acetonitrile (according to the recommendations provided in standard certificates) included 3- and 15-acetyl-deoxynivalenol (3-ADON and 15-ADON), enniatin A (Enn-A), enniatin A1 (Enn-A1), enniatin B (Enn-B) and enniatin B (Enn-B1). The initial concentration of each of the standards was 100 µg·mL^−1^. All standards were stored in amber glass vials maintained at approximately minus 20 °C. A mixture of all of the standards necessary for a particular analytical run was prepared immediately prior to the run. To prepare a mixture, specific volumes of individual standard solutions were transferred to a 5-mL volumetric flask. The flask was filled up to the mark with LC-MS-grade acetonitrile and thoroughly shaken. The contents were then transferred to an amber glass vial and stored at approximately minus 20 °C. The mixtures were used both to validate the method and to analyze the samples. The final concentrations (in μg·mL^−1^) of individual mycotoxin standards were as follows: AFB_1_, AFB_2_, AFG_1_ and AFG_2_: 0.08; Enn-A, Enn-A1, Enn-B, Enn-B1, OTA and OTB: 0.2; HFB_1_: 0.25; α-/β-ZOL and DAS: 0.4; HT-2, T-2 and NEO: 1; and FB_1_, FB_2_, FB_3_, DON-3G, 3-/15-ADON, ZEN, DON and FUS-X: 2. Some ^13^C-labelled internal standards were also used (ZEN, DAS, HT-2, FB_1_: 25; FB_2_, FB_3_: 10).

### 4.3. Sample Preparation

The samples were generally prepared in line with the QuEChERS (quick, easy, cheap, effective, rugged, safe) procedure proposed by Koesukwiwat *et al.* [13]. However, we modified the procedure to analyze the mycotoxins in the grain. Specifically, 2 g of dry mass of a ground sample placed in a falcon-type test tube were supplemented with ^13^C-labelled internal standards (ZEN 10 µL, HT2 10 µL, DAS 5 µL, FB_1_ 10 µL), 2 mL of water and 10 mL of 10% formic acid in acetonitrile. The tube contents were homogenized in a homogenizer for 3 min. Then, 2 g of anhydrous magnesium sulfate, 0.5 g of sodium chloride, 0.5 g of sodium citrate dihydrate and 0.25 g of sodium citrate dibasic sesquihydrate were added, after which the tubes were immediately energetically shaken and centrifuged (10,730× *g* for 10 min). The extract in acetonitrile was transferred to another 15-mL falcon test tube and extracted with hexane (5 mL) to remove the lipid fraction. The defatted extract was deep-frozen (at −30 °C) for approximately 24 h. Three hours prior to the end of the freezing period, a 5-mL cartridge with some glass wool in the bottom was also placed in the same freezer. The frozen supernatant was filtered into a 15-mL falcon centrifuge vial. The extract was supplemented with 1 g of anhydrous magnesium sulfate, 0.25 g of C-18 silica gel, 0.25 g of neutral alumina and 0.4 g of PSA. The samples were shaken and centrifuged for 5 min (10,730× *g*). Then, 2.5 mL of the supernatant were transferred to a reaction vial and evaporated in a stream of nitrogen flowing through a heater. The extract was dissolved in 300 µL of methanol and subsequently supplemented with 200 µL of HPLC-grade water. The solution was filtered through a nylon syringe filter with a 0.2-µm mesh size.

### 4.4. UHPLC-HRMS

An Acquity H-Class ultra-high performance liquid chromatograph coupled to an LCQ Premiere XE time-of-flight high-resolution mass spectrometer equipped with a UPLC C18 Cortecs 2.1 × 100 mm 1.6-µm column and a pre-column was used (Waters, Milford, MA, USA) as follows. Mobile Phase A: methanol and 0.2% formic acid; Phase B: water, 0.2% formic acid, 2 mM ammonium formate. Flow rate: 0.4 mL·min^−1^. Gradient of Phase A: from 1%–50% for 0–8 minutes, from 50%–95% for 8–11 min, constant at 95% for 11–22 min, from 95% down to 1% for 22–22.6 min and constant at 1% for 22.6–27 min. Sample volume injected into the column: 2.5 μL. Ion source: ESI-type at both positive and negative polarization. Ion source temperature: 150 °C, de-solvation temperature: 350 °C. Gaseous nitrogen flow rate: 750 L·min^−1^, cone flow rate: 20 L·min^−1^. Capillary bias: 3000 V/2800 V in positive and negative ion modes, respectively. The ion optics were operated in the V mode. The mass spectrometer was calibrated using solutions of the leucine encephalin solution. The precursor ions for each of the 26 analyzed mycotoxins are specified in Table S1.

### 4.5. Method Validation

To correct the matrix effects (which may reduce sensitivity), matrix matched calibration standards were used for quantification. Calibration standards were dissolved in the sample matrix prepared in the same manner as other samples. The material used for standard calibration preparation was mycotoxin-free. A diluted mixture of the standards and internal standards was added before the matrix samples were finally dissolved in methanol (300 μL) and water (200 μL). The concentrations of the internal standards in the matrix samples corresponded to the concentration of the standards in the samples waiting to be analyzed. The linearity of the individual compounds was evaluated in the following a concentration ranges (in µg·kg^−1^): DON, at 25–670; DON-3G, at 20–840; FUS-X, at 13–1020; NEO, at 2–402; 3- and 15-ADON, at 20–1,700; DAS, at 1–402; AFB_1_; AFB_2_; AFG_1_ and AFG_2_, at 4–70; HT-2 and T-2 toxin, at 1–670; FB_1_; FB_2_ and FB_3_, at 25–330; OTA and OTB, at 4–180; ZEN, at 2–670; α- and ß-ZOL, at 2–200; and Enn-A; Enn-A1; Enn-B and Enn-B1, at 1–13.

The limits of quantification (LOQs) of the individual mycotoxins were determined as the concentrations at which the signal-to-noise ratio decreased to 10:1. The wheat and triticale matrices were similar in terms of practically identical LOQs for a given mycotoxin (different values for various mycotoxins). Similarly, the barley and oat matrices were similar in terms of the practically identical LOQs for a given mycotoxin (different values for various mycotoxins and often different from the corresponding values obtained for wheat/triticale). An overwhelming majority of 100 coefficients of correlation (25 analytes × 4 matrices) was above 0.99 with a few exceptions, where values ranged between 0.98 and 0.99 (the worst case: 0.9774). The LOQs and correlation coefficients obtained for the individual mycotoxins in wheat, triticale, barley and oats are shown in Table S1.

Matrix samples (2 g) spiked with certain mycotoxin standards at three levels were used to measure the recovery rates (R%) and method repeatability (precision, RSD%). The results are shown in Table S2. For the majority of the mycotoxins, the recovery rates ranged between 70% and 120% (depending on the matrix), whereas the RSD values did not exceed 30%. The exceptions included: DON-3G (R = 41%–58%, RSD = 2%–21%); FB_1_ (41%–74% and 4%–18%, respectively); FB_2_ (63%–91% and 4%–18%, respectively); and FB_3_ (60%–89% and 4%–19%, respectively). Indeed, the measured recovery rates were used to calculate the unknown concentrations of the analytes.

The developed method was also assessed within the framework of certain FAPAS (Food Analysis Performance Assessment Scheme) and Romer Labs inter-laboratory proficiency tests. As observed in Table S3, the obtained *z*-score values fell within the –1.3–+1.2 range depending on the analyte and the matrix and were considered satisfactory in all cases.

### 4.6. Statistical Procedure

Only samples contaminated above the LOQ level were included in calculations of average and/or median values. The experimental data were statistically evaluated using the Statgraphics 4.1 software package (Graphics Software System, STCC, Inc., Rockville, MD, USA). A one-way ANOVA was used to assess the significance of the differences between the determined mycotoxin concentrations. Tukey's test at α = 0.05 was used for the paired tests.

## Figures and Tables

**Table 1:** Concentrations (µg·kg^−1^) of mycotoxins found in samples of winter wheat cultivated in five regions of Poland.

Chemical Name	Żelisławki (*n* = 18)					Wielichowo (*n* = 36)					Osiny (*n* = 18)					Borusowa (*n* = 18)					Werbkowice (*n* = 9)				
	Positive Samples	AVG ^1^	Median	Min	Max	Positive Samples	AVG	Median	Min	Max	Positive Samples	AVG	Median	Min	Max	Positive Samples	AVG	Median	Min	Max	Positive Samples	AVG	Median	Min	Max
		µg·kg^−1^					µg·kg^−1^					µg·kg^−1^					µg·kg^−1^					µg·kg^−1^			
ZEN	0	-	-	-	-	2	4	4	3	4	18	16	8	1	45	18	43	40	7	100	9	9	9	2	15
α-ZOL	0	-	-	-	-	0	-	-	-	-	0	-	-	-	-	0	-	-	-	-	1	8	8	8	8
β-ZOL	0	-	-	-	-	0	-	-	-	-	8	12	10	5	24	18	7	7	2	21	4	6	7	5	7
HT-2 toxin	0	-	-	-	-	0	-	-	-	-	9	15	5	2	55	3	7	6	5	9	6	6	6	4	8
T-2 toxin	0	-	-	-	-	0	-	-	-	-	16	3	2	1	22	18	2	2	1	6	7	4	4	3	5
DON	0	-	-	-	-	1	25	25	25	25	18	590	606	82	1616	18	960	678	209	2975	9	762	694	277	1721
3-/15-ADON	0	-	-	-	-	0	-	-	-	-	15	32	30	25	48	13	59	53	32	98	6	51	45	26	85
DON-3G	0	-	-	-	-	0	-	-	-	-	8	106	116	43	166	12	74	52	40	218	7	118	77	40	356
FUS-X	0	-	-	-	-	0	-	-	-	-	0	-	-	-	-	0	-	-	-	-	0	-	-	-	-
DAS	0	-	-	-	-	0	-	-	-	-	0	-	-	-	-	0	-	-	-	-	0	-	-	-	-
NEO	0	-	-	-	-	0	-	-	-	-	1	2	2	2	2	15	11	11	2	32	0	-	-	-	-
AFB_1_	0	-	-	-	-	0	-	-	-	-	0	-	-	-	-	0	-	-	-	-	0	-	-	-	-
AFB_2_	0	-	-	-	-	0	-	-	-	-	0	-	-	-	-	0	-	-	-	-	0	-	-	-	-
AFG_1_	0	-	-	-	-	0	-	-	-	-	0	-	-	-	-	0	-	-	-	-	0	-	-	-	-
AFG_2_	0	-	-	-	-	0	-	-	-	-	0	-	-	-	-	0	-	-	-	-	0	-	-	-	-
OTA	0	-	-	-	-	0	-	-	-	-	1	4	4	4	4	0	-	-	-	-	1	7	7	7	7
OTB	0	-	-	-	-	0	-	-	-	-	0	-	-	-	-	0	-	-	-	-	0	-	-	-	-
FB_1_	0	-	-	-	-	0	-	-	-	-	0	-	-	-	-	4	68	40	40	150	1	50	50	50	50
FB_2_	0	-	-	-	-	0	-	-	-	-	0	-	-	-	-	0	-	-	-	-	0	-	-	-	-
FB_3_	0	-	-	-	-	0	-	-	-	-	0	-	-	-	-	0	-	-	-	-	0	-	-	-	-
HFB_1_	0	-	-	-	-	0	-	-	-	-	0	-	-	-	-	0	-	-	-	-	0	-	-	-	-
Enn-A	0	-	-	-	-	2	1	1	1	1	10	3	3	1	13	18	8	5	1	23	6	3	3	1	8
Enn-A1	0	-	-	-	-	4	4	4	2	5	16	8	7	3	18	18	12	10	1	28	9	5	5	1	10
Enn-B	18	2	2	1	7	30	18	6	1	156	18	84	76	39	142	18	275	179	20	1,981	9	651	695	164	982
Enn-B1	15	2	1	1	3	23	4	2	1	22	18	38	30	15	68	18	89	67	8	368	9	68	80	13	97

^1^ AVG, average of positive samples.

**Table 2:** Concentrations (µg·kg^−1^) of mycotoxins found in investigated samples of grain other than winter wheat.

Grain Variety Mycotoxin	Spring Barley (*n* = 8)					Winter Barley (*n* = 16)					Oats (*n* = 4)					Triticale (*n* = 20)				
	Positive Samples	AVG ^1^	Median	Min	Max	Positive Samples	AVG	Median	Min	Max	Positive Samples	AVG	Median	Min	Max	Positive Samples	AVG	Median	Min	Max
		µg·kg^−1^					µg·kg^−1^					µg·kg^−1^					µg·kg^−1^			
ZEN	6	13	10	2	31	10	7	7	2	19	4	10	11	5	15	20	29	23	4	86
α-ZOL	0	-	-	-	-	0	-	-	-	-	0	-	-	-	-	0	-	-	-	-
β-ZOL	1	8	8	8	8	0	-	-	-	-	2	8	8	6	9	0	-	-	-	-
HT-2 toxin	8	26	21	8	74	12	11	5	3	69	4	69	68	46	93	2	8	8	2	13
T-2 toxin	8	5	5	2	11	7	2	2	1	5	4	22	25	9	29	2	3	3	2	3
DON	4	138	126	76	222	16	602	512	54	1602	4	113	118	67	149	20	573	511	196	1326
3-/15-ADON	1	62	62	62	62	0	-	-	-	-	0	-	-	-	-	20	162	134	36	374
DON-3G	1	58	58	58	58	6	146	115	43	277	0	-	-	-	-	15	138	77	40	434
FUS-X	0	-	-	-	-	0	-	-	-	-	4	64	64	47	81	0	-	-	-	-
DAS	0	-	-	-	-	0	-	-	-	-	4	2	2	1	2	0	-	-	-	-
NEO	0	-	-	-	-	7	16	15	5	36	0	-	-	-	-	2	6	6	6	6
AFB_1_	0	-	-	-	-	0	-	-	-	-	0	-	-	-	-	0	-	-	-	-
AFB_2_	0	-	-	-	-	0	-	-	-	-	0	-	-	-	-	0	-	-	-	-
AFG_1_	0	-	-	-	-	0	-	-	-	-	0	-	-	-	-	0	-	-	-	-
AFG_2_	0	-	-	-	-	0	-	-	-	-	0	-	-	-	-	0	-	-	-	-
OTA	0	-	-	-	-	0	-	-	-	-	0	-	-	-	-	1	8	8	8	8
OTB	0	-	-	-	-	0	-	-	-	-	0	-	-	-	-	1	7	7	7	7
FB_1_	1	101	101	101	101	0	-	-	-	-	0	-	-	-	-	1	342	342	342	342
FB_2_	0	-	-	-	-	0	-	-	-	-	0	-	-	-	-	1	151	151	151	151
FB_3_	0	-	-	-	-	0	-	-	-	-	0	-	-	-	-	1	113	113	113	113
HFB_1_	0	-	-	-	-	0	-	-	-	-	0	-	-	-	-	0	-	-	-	-
Enn-A	3	2	2	1	3	0	-	-	-	-	0	-	-	-	-	14	32	23	8	135
Enn-A1	8	21	20	9	45	16	6	4	1	16	4	6	5	2	11	20	357	274	67	882
Enn-B	8	197	195	115	301	16	150	140	49	253	4	117	132	42	162	20	1360	1258	473	3328
Enn-B1	8	119	108	66	240	16	46	45	8	81	4	41	40	15	67	20	552	415	149	1347

^1^ AVG, average of positive samples.

**Table 3:** Distribution of cereal crops in different locations.

Species	Location				
	Osiny	Żelisławki	Wielichowo	Borusowa	Werbkowice
Winter cereals (number of samples)					
Wheat	18	18	36	18	9
Triticale	20	-	-	-	-
Barley	16	-	-	-	-
Spring cereals (number of samples)					
Barley	8	-	-	-	-
Oats	4	-	-	-	-

## Supplementary Materials

The following are available online at www.mdpi.com/2072-6651/8/6/160/s1. Table S1: Limits of quantification (LOQ) (µg·kg^−1^) and coefficients of correlation (*r*) obtained for wheat, triticale, barley and oats. Table S2: Low, middle and high values of the recovery rates and RSD for the investigated mycotoxins in the investigated matrices. Table S3: Results of some inter-laboratory proficiency tests (organized in 2014–2015), in which the developed method was assessed (selected mycotoxins in selected grain matrices).

## Abbreviations

The following abbreviations are used in this manuscript:

FHB	Fusarium head blight
DON	deoxynivalenol
DON-3G	deoxynivalenol-3-glucoside
FUS-X	fusarenon X
NEO	neosolaniol
3-ADON	3-acetyl-deoxynivalenol
15-ADON	15-acetyl-deoxynivalenol
DAS	diacetoxyscirpenol
HT-2	HT-2 toxin
T-2	T-2 toxin
AFB_1_	aflatoxin B_1_
AFB_2_	aflatoxin B_2_
AFG_1_	aflatoxin G_1_
AFG_2_	aflatoxin G_2_
AFs	aflatoxins
FB_1_	fumonisin B_1_
FB_2_	fumonisin B_2_
FB_3_	fumonisin B_3_
HFB_1_	hydrolysed fumonisin B_1_
OTA	ochratoxin A
OTB	ochratoxin B
ZEN	zearalenone
α-ZOL	α-zearalenol
β-ZOL	β-zearalenol
Enn-A	enniatin A
Enn-A1	enniatin A-1
Enn-B	enniatin B
Enn-B1	enniatin B-1
Enn(s)	enniatins
LOQ	limit of quantification
AVG	average
RH	relative humidity
