# Supplementary material for: Occurrence of 26 Mycotoxins in the Grain of Cereals Cultivated in Poland
Source: Toxins (Basel). 2016 May 25;8(6):160. doi: 10.3390/toxins8060160 (PMC4926127; doi:10.3390/toxins8060160)
Supplement: Supplementary file 1 [file toxins-08-00160-s001.pdf]

Supplementary Materials: Occurence of 26 Mycotoxins in Grain of Cereals Cultivated in Poland

Marcin Bryła, Agnieszka Waśkiewicz, Grażyna Podolska, Krystyna Szymczyk, Renata Jędrzejczak, Krzysztof Damaziak and Alicja Sułek

Table S1. Limits of quantification (LOQ) (µg·kg^{-1}) and coefficients of correlation (*r*) obtained for wheat, triticale, barley and oats.

Mycotoxin	Precursor Ion (m/z)	Retention Time tr (min)	Internal Standard	Wheat		Triticale		Barley		Oats	
				LOQ	*r*	LOQ	*r*	LOQ	*r*	LOQ	*r*
DON	319.02 $[M + Na]^+$, 297.08 $[M + H]^+$	3.72	^{13}C-DAS	25	0.9923	25	0.9821	40	0.9823	40	0.9911
DON-3G	481.15 $[M + Na]^+$	3.94	^{13}C-DAS	30	0.9917	30	0.9931	40	0.9816	40	0.9963
FUS-X	377.12 $[M + Na]^+$, 372.13 $[M + NH_4]^+$	4.91	^{13}C-DAS	13	0.9905	13	0.9899	20	0.9929	20	0.9819
NEO	405.04 $[M + Na]^+$, 400.08 $[M + NH_4]^+$	5.39	^{13}C-DAS	2	0.9935	2	0.9977	9	0.9857	9	0.9922
3-ADON, 15-ADON	361.06 $[M + Na]^+$	5.89	^{13}C-DAS	20	0.9983	20	0.9877	30	0.9774	30	0.9864
DAS	389.11 $[M + Na]^+$, 384.12 $[M + NH_4]^+$	8.54	^{13}C-DAS	1	0.9934	1	0.9974	1	0.9811	1	0.9953
HT-2	447.18 $[M + Na]^+$, 442.11 $[M + NH_4]^+$	9.79	^{13}C-HT-2	2	0.9989	2	0.9881	2	0.9844	2	0.9799
T-2	489.13 $[M + Na]^+$, 484.12 $[M + NH_4]^+$	10.41	^{13}C-HT-2	1	0.9863	1	0.9847	1	0.9898	1	0.9891
AFB_1	313.09 $[M + H]^+$	6.99	^{13}C-HT-2	4	0.9956	4	0.9959	5	0.9974	5	0.9941
AFB_2	315.11 $[M + H]^+$	7.37	^{13}C-HT-2	4	0.9876	4	0.9986	5	0.9881	5	0.9977
AFG_1	329.12 $[M + H]^+$	7.77	^{13}C-HT-2	4	0.9934	4	0.9968	5	0.9887	5	0.9846
AFG_2	331.11 $[M + H]^+$	8.15	^{13}C-HT-2	4	0.9931	4	0.9891	5	0.9991	5	0.9908
FB_1	722.43 $[M + H]^+$	10.03	^{13}C-FB1	50	0.9988	50	0.9965	65	0.9899	65	0.9931
FB_2	706.41 $[M + H]^+$	10.75	^{13}C-FB2	25	0.9975	25	0.9895	40	0.9974	40	0.9844
FB_3	706.41 $[M + H]^+$	10.47	^{13}C-FB3	25	0.9970	25	0.9947	40	0.9849	40	0.9976
HFB_1	406.10 $[M + H]^+$	9.89	^{13}C-DAS	6	0.9932	6	0.9955	12	0.9967	12	0.9913
OTA	404.11 $[M + H]^+$, 402.07 $[M - H]^-$	10.68	^{13}C-DAS or ^{13}C-ZEN	4	0.9950	4	0.9895	7	0.9996	7	0.9901
OTB	370.09 $[M + Na]^+$	10.12	^{13}C-DAS	4	0.9951	4	0.9963	7	0.9971	7	0.9890
ZEN	317.10 $[M - H]^-$	10.54	^{13}C-ZEN	2	0.9928	2	0.9947	2	0.9963	2	0.9936
α-ZOL	319.12 $[M - H]^-$	10.40	^{13}C-ZEN	2	0.9970	2	0.9991	2	0.9932	2	0.9932
β-ZOL	319.12 $[M - H]^-$	9.94	^{13}C-ZEN	2	0.9917	2	0.9985	2	0.9910	2	0.9899
Enn-A	699.31 $[M + NH_4]^+$	12.25	^{13}C-DAS	1	0.9972	1	0.9999	1	0.9941	1	0.9964
Enn-A1	685.39 $[M + NH_4]^+$	12.37	^{13}C-DAS	1	0.9991	1	0.9989	1	0.9913	1	0.9879
Enn-B	657.32 $[M + NH_4]^+$	12.00	^{13}C-DAS	1	0.9977	1	0.9871	1	0.9842	1	0.9997
Enn-B1	671.31 $[M + NH_4]^+$	12.14	^{13}C-DAS	1	0.9978	1	0.9921	1	0.9960	1	0.9876

Table S2. Low, Middle and High values of recovery (R) rates & relative standard deviation (RSD) for the investigated mycotoxins in the investigated matrices.

Matrix / Mycotoxin	Wheat						Triticale						Oats						Barley					
	Low	RSD	Middle	RSD	High	RSD	Low	RSD	Middle	RSD	High	RSD	Low	RSD	Middle	RSD	High	RSD	Low	RSD	Middle	RSD	High	RSD
	%																							
DON [a]	103	7.4	103	3.5	104	4.2	79	2.4	93	9.2	87	10.3	116	3.4	100	16.9	104	12.9	82	13.0	105	11.0	99	17.3
DON-3G [b]	47	17.5	58	2.0	64	9.2	56	3.9	48	11.9	48	9.4	58	10.6	52	20.0	50	18.1	41.2	14.7	56	21.3	48	10.6
FUS-X [a]	86	9.4	115	12.5	102	2.9	88	2.6	92	6.8	93	8.4	111	13.5	117	11.5	94	9.7	117	8.2	109	7.9	80	18.4
NEO [c]	102	10.7	96	13.3	93	3.2	88	22.0	92	5.9	95	4.1	109	8.3	115	6.1	95	10.1	93	18.5	110	6.4	83	10.3
Sum of 3-and 15-ADON [a]	98	18.6	115	7.1	102	10.6	83	15.5	102	5.7	93	12.2	107	5.7	89	16.3	92	12.1	89	19.7	90	6.0	103	15.2
DAS [d]	110	16.6	106	13.5	109	11.1	95	11.8	100	9.8	106	17.0	114	5.4	116	5.6	94	5.9	114	16.2	115	1.1	106	5.5
HT-2 Toxin [c]	91	10.7	115	5.4	109	5.9	85	6.2	92	9.9	112	6.4	96	7.9	116	13.7	95	6.5	100	16.5	113	7.3	103	11.3
T-2 Toxin [c]	79	10.2	111	8.4	107	15.4	93	9.1	85	6.1	121	5.5	116	5.8	104	17.3	87	11.8	109	4.1	118	9.4	108	14.0
FB_1 [a]	56	15.3	46	17.1	58	13.0	59	15.1	41	10.2	53	6.8	54	16.3	52	4.4	70	14.0	74	13.3	62	5.1	58	18.0
FB_2 [a]	67	16.6	78	5.1	63	3.9	91	15.2	89	16.3	89	9.5	72	16.4	67	17.9	75	14.6	72	9.2	64	12.1	77	6.1
FB_3 [a]	67	14.4	82	10.2	72	7.6	60	14.8	89	12.8	80	8.9	80	14.4	75	18.7	70	15.8	66	10.7	71	4.3	80	10.3
HFB_1 [e]	69	11.0	46	17.1	75	6.1	85	5.5	77	5.3	63	10.5	76	11.5	85	22.2	103	8.6	78	8.7	65	11.8	92	16.1
OTA [f]	110	3.4	104	12.4	100	12.4	99	13.0	92	13.8	108	17.1	100	7.6	113	7.7	119	6.7	116	14.0	111	3.9	96	12.5
OTB [f]	122	14.8	122	5.9	110	9.4	124	13.4	115	5.5	119	7.7	107	7.9	111	7.6	117	6.8	118	16.6	117	11.5	101	8.9
AFB_1 [g]	85	7.6	93	9.4	79	10.3	73	8.1	94	3.8	72	10.1	108	10.8	92	7.5	75	10.6	71	13.8	71	17.4	96	8.8
AFB_2 [g]	116	8.9	117	9.7	105	4.5	95	6.5	107	9.8	99	12.6	102	8.3	104	14.0	88	9.0	114	11.1	119	4.5	89	19.3
AFG_1 [g]	113	9.4	114	9.2	93	7.3	85	5.3	93	10.6	75	11.2	84	22.0	100	6.7	86	15.0	98	17.0	104	16.5	74	11.2
AFG_2 [g]	115	10.5	114	14.6	99	16.2	89	3.0	103	12.1	106	9.1	93	11.2	108	14.3	98	9.0	106	11.2	97	13.1	109	7.0
Enn-A [f]	118	16.1	87	8.1	114	5.7	94	8.3	88	8.2	88	9.1	93	11.2	108	14.3	98	9.0	88	16.2	119	14.1	87	17.0
Enn-A1 [f]	124	17.0	105	13.1	113	4.8	87	15.3	78	16.8	84	13.8	102	5.6	72	12.9	99	11.9	119	10.3	111	9.5	103	16.4
Enn-B [f]	104	7.4	109	4.4	108	8.6	90	15.0	92	13.6	82	9.1	100	13.5	111	10.6	101	10.8	119	8.8	112	6.7	116	12.2
Enn-B1 [f]	102	12.7	108	4.9	107	5.6	85	14.5	83	7.2	80	12.2	109	12.4	90	7.5	101	8.9	107	18.5	117	6.7	103	14.7
ZEN [a]	101	6.0	117	5.4	117	5.4	78	13.6	108	7.2	110	11.4	97	15.2	116	3.2	123	4.2	94	6.3	105	2.5	91	5.3
α-ZOL [d]	86	19.2	94	14.0	114	10.7	100	24.0	94	8.7	103	10.8	83	10.9	112	4.9	104	13.9	65	10.6	78	4.3	106	9.0
ß-ZOL [d]	91	18.7	89	7.3	103	9.3	72	16.0	109	6.3	99	13.8	92	10.0	101	10.3	106	3.0	67	17.4	84	12.9	98	8.9

Spiking levels: [a] low = 200 $\mu g \cdot kg^{-1}$; middle = 250 $\mu g \cdot kg^{-1}$; high = 300 $\mu g \cdot kg^{-1}$; [b] low = 50 $\mu g \cdot kg^{-1}$; middle = 62.5 $\mu g \cdot kg^{-1}$; high = 75 $\mu g \cdot kg^{-1}$; [c] low = 100 $\mu g \cdot kg^{-1}$; middle = 125 $\mu g \cdot kg^{-1}$; high = 150 $\mu g \cdot kg^{-1}$; [d] low = 40 $\mu g \cdot kg^{-1}$; middle = 50 $\mu g \cdot kg^{-1}$; high = 60 $\mu g \cdot kg^{-1}$; [e] low = 25 $\mu g \cdot kg^{-1}$; middle = 31,25 $\mu g \cdot kg^{-1}$; high = 38 $\mu g \cdot kg^{-1}$; [f] low = 20 $\mu g \cdot kg^{-1}$; middle = 25 $\mu g \cdot kg^{-1}$; high = 30 $\mu g \cdot kg^{-1}$; [g] low = 4 $\mu g \cdot kg^{-1}$; middle = 5 $\mu g \cdot kg^{-1}$; high = 6 $\mu g \cdot kg^{-1}$.

Table S3. Results of some inter-laboratory proficiency tests (organized in 2014–2015), in which the developed method was assessed (selected mycotoxins in selected grain matrices).

Proficiency Test	Compound	Matrix	Assigned Value (μg·kg^{-1})	Determined Value (μg·kg^{-1})	*z*-Score Value	Result
Romer Labs CSSMY006-M14421Z (2014)	ZEN [1]	Wheat	119	133	0.5	satisfactory
FAPAS 04260 (2015)	AFB_1 [2]	Maize	5.09	5.43	0.3	satisfactory
	AFs (Total) [3]		5.21	5.43	0.4	satisfactory
	DON [4]		1191	946	–1.3	satisfactory
	OTA [5]		3.29	4.25	1.3	satisfactory
	ZEN		200	221	0.5	satisfactory
FAPAS 22116 (2015)	T-2 [6]	Animal feed	280	330	0.9	satisfactory
	HT-2 [7]		411	502	1.2	satisfactory
	T-2 + HT-2		693	832	1.2	satisfactory
Romer Labs CSSMY008-M15421A (2015)	AFB_1	Maize	6.0	5.46	–0.4	satisfactory
	AFs (Total)		6.4	6.03	–0.3	satisfactory

[1] ZEN—zearalenone, [2] AFB_1—B_1 aflatoxin, [3] AFs (Total)—sum of aflatoxins, [4] DON—deoxynivalenol, [5] OTA—ochratoxin A, [6] T-2—T-2 toxin, [7] HT-2—HT-2 toxin
